# Epigallocatechin‐3‐gallate improves the quality of maternally aged oocytes

**DOI:** 10.1111/cpr.13575

**Published:** 2023-11-27

**Authors:** HongHui Zhang, Wei Su, RuSong Zhao, Mei Li, ShiGang Zhao, Zi‐Jiang Chen, Han Zhao

**Affiliations:** ^1^ State Key Laboratory of Reproductive Medicine and Offspring Health Shandong University Jinan China; ^2^ The Affiliated Suzhou Hospital of Nanjing Medical University, Suzhou Municipal Hospital, Gusu School Nanjing Medical University Nanjing China; ^3^ Key Laboratory of Reproductive Endocrinology of Ministry of Education Shandong University Jinan China; ^4^ National Research Center for Assisted Reproductive Technology and Reproductive Genetic Shandong University Jinan China; ^5^ Research Unit of Gametogenesis and Health of ART‐Offspring, Chinese Academy of Medical Sciences (No.2021RU001) Jinan China; ^6^ Shandong Key Laboratory of Reproductive Medicine, Shandong Provincial Hospital Affiliated to Shandong First Medical University Jinan China; ^7^ Shanghai Key Laboratory for Assisted Reproduction and Reproductive Genetics Shanghai China; ^8^ Center for Reproductive Medicine, Ren Ji Hospital, School of Medicine Shanghai Jiao Tong University Shanghai China

## Abstract

The decline in female fertility as age advances is intricately linked to the diminished developmental potential of oocytes. Despite this challenge, the strategies available to enhance the quality of aged oocytes remain limited. Epigallocatechin‐3‐gallate (EGCG), characterised by its anti‐inflammatory, antioxidant and tissue protective properties, holds promise as a candidate for improving the quality of maternally aged oocytes. In this study, we explored the precise impact and underlying mechanisms of EGCG on aged oocytes. EGCG exhibited the capacity to enhance the quality of aged oocytes both in vitro and in vivo. Specifically, the application of EGCG in vitro resulted in noteworthy improvements, including an increased rate of first polar body extrusion, enhanced mitochondrial function, refined spindle morphology and a reduction in oxidative stress. These beneficial effects were further validated by the improved fertility observed among aged mice. In addition, our findings propose that EGCG might augment the expression of Arf6. This augmentation, in turn, contributes to the assembly of spindle‐associated F‐actin, which can contribute to mitigate the aneuploidy induced by the disruption of spindle F‐actin within aged oocytes. This work thus contributes not only to understanding the role of EGCG in bolstering oocyte health, but also underscores its potential as a therapeutic intervention to address fertility challenges associated with advanced age.

## Graphical abstract

This study delves into the precise impact and underlying mechanisms of EGCG on aged oocytes. Both in vitro and in vivo experiments demonstrate EGCG's capacity to enhance the quality of aged oocytes. In vitro application of EGCG yields remarkable improvements, including heightened rates of first polar body extrusion, refined spindle morphology, enhanced mitochondrial function, and reduced oxidative stress. Besides, EGCG may enhance Arf6 expression through direct interaction in aged oocytes, consequently promoting the assembly of spindle‐associated F‐actin and mitigating the aneuploidy induced by the disruption of spindle F‐actin. In addition, these positive outcomes are corroborated by enhanced fertility observed in aged mice, providing a therapeutic intervention to address fertility challenges associated with advanced age.

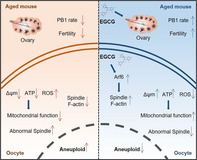

## 1 INTRODUCTION

In numerous countries, there is a growing trend among couples to embark on at later stages of life.^1^, ^2^, ^3^ As is widely recognised, the decline in female fertility becomes notably pronounced after around the age of the early 30s and the occurrence of pregnancy beyond the age of 45 is rare.^4^, ^5^ The decrease in female fertility as age advances can be primarily attributed to a substantial reduction in the quantity and quality of follicles and oocytes.^4^, ^6^, ^7^, ^8^ This decline is often accompanied by a notable increase in the occurrence of infertility, miscarriages, embryo‐related complications and congenital birth anomalies.^4^, ^9^ Despite the critical nature of this issue, there has been a lack of comprehensive exploration into effective strategies aimed at preserving oocyte quality as women age.

Oxidative stress plays a significant role in the ageing of oocytes, stemming from an imbalance between prooxidants and antioxidants within a biological system, resulting in an excess of reactive oxygen species (ROS).^8^, ^10^, ^11^ These ROS are predominantly generated during the process of oxygen consumption by the mitochondrial respiratory chain. Intriguingly, mitochondria, while being central to energy production, are also susceptible to damage from ROS, which adds to the constellation of challenges affecting reproductive function in ageing females.^8^, ^12^, ^13^ The influence of ROS is multi‐faceted. On one hand, it can lead to meiotic arrest and an increase in degenerated oocytes.^14^ Furthermore, oxidative stress can trigger apoptosis in zygotes and exert a pronounced impact on embryo development.^15^, ^16^ Additionally, ROS can compromise the integrity of the spindle assembly checkpoint in ageing oocytes, thereby exacerbating errors in chromosome segregation and contributing to age‐related aneuploidy.^17^

Epigallocatechin‐3‐gallate (EGCG), the most abundant polyphenol in green tea, boasts an array of characteristics, including anti‐inflammatory, anti‐oxidation, anti‐fibrosis and tissue protective properties.^18^ Its role as an antioxidant has prompted extensive researches into its therapeutic potential across various diseases, including within the female reproductive system.^19^ EGCG's antioxidant activity has shown promise in mitigating oxidative stress‐related infertility.^20^ For instance, the administration of EGCG at a lower concentration significantly improved the oocyte maturation and blastocyst development in bovine,^21^, ^22^ porcine^23^ and mice.^24^ While recent research has highlighted the potential benefits of EGCG in safeguarding porcine oocytes from post‐ovulatory ageing by counteracting oxidative stress,^25^ the comprehensive understanding of EGCG's involvement in the maturation and development of aged oocytes is still limited. Further exploration is needed to unravel the extent of EGCG's impact on aged oocytes and the underlying mechanisms that drive its effects in enhancing their maturation and developmental quality.

In this study, we sought to investigate the potential of EGCG to enhance fertility in aged mice. Our results revealed that EGCG treatment improved aged oocyte quality, leading to increase in vitro and in vivo maturation rates. Specially, EGCG supplementation effectively reduced intracellular ROS, enhanced mitochondrial function, and preserved the normal morphology of oocyte spindles. In addition, EGCG possesses the capacity to potentially regulate spindle‐associated F‐actin expression through Arf6 regulation, thereby potentially alleviating aneuploidy induced by the disruption of spindle‐associated F‐actin in aged oocytes.

## 2 MATERIALS AND METHODS

### 2.1 In vivo treatment of EGCG

Female ICR mice aged 9–10 months (Beijing Vital River Laboratory Animal Technology Co., Ltd.) were randomly assigned to five distinct groups. Subsequently, freshly‐prepared EGCG was administered via intraperitoneal injections for six consecutive days.

To assess in‐vivo oocyte maturation, superovulation was initiated concurrently on the fourth day of EGCG injection. This procedure involved the administration of 15 IU of pregnant mare's serum gonadotropin (PMSG, NINGBO SANSHENG), followed by 15 IU of human chorionic gonadotropin (HCG, NINGBO SANSHENG) after 46–48 h. Approximately 16–18 h later, the mice were sacrificed via cervical dislocation, and cumulus‐oocyte complexes were collected from the ampulla of the fallopian tube. Employing hyaluronidase (Sigma) digestion, granulosa cells were removed to assess the influence of varying EGCG concentrations on the quantity and quality of metaphase II (MII) oocytes. For fertility assessment, following 6 days of EGCG treatment, female mice were introduced to fertile male mice and allowed to mate for a duration of 3 months.

### 2.2 In vitro maturation and fertilisation of mouse oocytes

For in vitro maturation, healthy female ICR mice, aged 9–10 months, were selected as a representative model of natural ageing. In order to obtain germinal vesicle (GV) oocytes, the ageing mice were subjected to intraperitoneal injections of 15 IU of pregnant mare's serum gonadotropin. After a span of 44 h, the collected GV oocytes were cultured in M16 medium (Sigma, USA) containing varying concentrations of EGCG. This culturing process was carried out under specific conditions, including 6% CO_2_ and 5% O_2_, at a temperature of 37°C for a duration of 16 h. The objective of this procedure was to determine the rates of germinal vesicle breakdown (GVBD) or first polar body (PB1) extrusion, respectively.

For in vitro fertilisation, young ICR female mice (4–6 weeks) were subjected to intraperitoneal injections of 5 IU of pregnant mare's serum gonadotropin, followed by 5 IU of human chorionic gonadotropin after 46–48 h. Sperm were collected in cauda epididymis from ICR male mice aged 8–12 weeks and were capacitated in G‐IVF plus medium (Vitrolife) for 1 h. Cumulus oocyte complexes (COC) were collected 16 h after HCG injection and the capacitated sperm were added together in G‐IVF plus medium covered with mineral oil. After cultured for 4–6 h at 37°C in 5% CO_2_ atmosphere, the formed fertilised eggs would be transferred to G1 plus medium (Vitrolife) supplemented with EGCG. The protocol for the animal study was reviewed and approved by the Institutional Review Board of Reproductive Medicine, Shandong University.

### 2.3 ROS assessment

To quantify ROS in oocytes, MII oocytes cultured with or without 10 μM EGCG in vitro were collected and incubated with 10 mM DCFH‐DA (Beyotime) in M16 medium at 37°C for 30 min. Following washing in fresh M16 medium, the oocytes were immediately imaged at the maximum section using a confocal microscope (Dragonfly, Andor Technology, UK) under the mode of time‐series and 488 nm excitation light. Fluorescence intensity of each oocyte was measured using Image J (National Institutes of Health, USA).

### 2.4 Mitochondrial distribution and membrane potential

Mitochondrial distribution was assessed by culturing the MII oocytes in M16 medium with 200 nM Mito‐Tracker (Beyotime) at 37°C for 30 min. Mitochondrial membrane potential (MMP) was determined by incubating oocytes in M16 medium containing 2 μM JC‐1 (Beyotime) at 37°C for 30 min. Immunofluorescence analysis was performed on control DMSO and EGCG‐treated oocytes under consistent conditions. After sufficient washing, the oocytes were examined under a laser scanning confocal microscope (Dragonfly, Andor Technology, UK) under the mode of time‐series at the maximum section, and then the mean fluorescence intensity was quantified using Image J.

### 2.5 Oocyte RNA extraction and real‐time PCR

Total RNA was extracted using the Dynabeads mRNA DIRECT™ Kit (ThermoFisher), and cDNA was synthesised using HiScript II Reverse Transcriptase (Vazyme) according to the manufacture's instructions. In brief, approximately 10 oocytes were lysed using 80 μL of Lysis Binding Buffer for approximately 3 min. The beads were then resuspended by pipetting, and 20 μL of beads were added to the lysis for 3–5 min at room temperature to facilitate the hybridisation of the mRNA's polyA tail with the oligo(dT) on the beads. Subsequently, the supernatant was removed using a magnet, followed by two washes with 150 μL of Washing Buffer A and one wash with Washing Buffer B, all at room temperature. Finally, 10 μL of 10 mM Tris–HCl (Elution Buffer) was added, and the mixture was incubated at 65–80°C for 2 min to elute the mRNA from the beads. For cDNA synthesis, the 10 μL elution buffer containing mRNA was combined with 4 μL of 4× gDNA Wiper mix and 2 μL of RNase‐free ddH2O, and incubated at 42°C for 2 min. This was followed by the addition of 4 μL of 5× HiScript II qRT SuperMix II (Vazyme) at 50°C for 15 min, and then at 85°C for 5 s.

For RT‐PCR, 4 μL cDNA template was mixed with 5 μL TB Green Premix Ex Taq (TaKaRa) and the mRNA expression was confirmed via real‐time quantitative PCR with the LightCycler 480 (Roche, Germany). GAPDH mRNA level served as the internal control, and Microsoft Excel was used for data analysis. Primer sequences are provided in Table S2.

### 2.6 Immunofluorescence staining of oocytes

Oocytes were fixed in 4% paraformaldehyde, permeabilised in phosphate‐buffered saline (PBS) with 0.5% Triton X‐100, blocked with 1% bovine serum albumin (BSA, Sigma) in PBS, and then incubated with primary antibody in 1% BSA overnight at 4°C. Spindle morphology was evaluated using anti‐α‐tubulin‐FITC antibody, and F‐actin was displayed by Rhodamine Phalloidin. Distribution and expression of ARF6 were analysed using ARF6 Antibody as the primary antibody. After washing, oocytes were incubated with secondary antibody (Invitrogen) for ARF6 staining. All the oocytes were stained with 4′,6‐diamidino‐2‐phenylindole (DAPI), and imaged by laser confocal microscopy. Immunofluorescence analysis was performed on control and EGCG‐treated oocytes under parallel conditions, with mean fluorescence intensity quantified using Image J. For the analysis of F‐actin signals, three regions of interest (ROI) of equal size in the oocyte's cortical, cytoplasmic, or spindle regions were randomly chosen. The fluorescence intensity values of these regions were measured and then averaged. Detailed information about the primary antibodies can be found in Table S3.

### 2.7 Determination of ATP content in oocytes

ATP content was assessed using a commercial assay kit based on a fluorescein‐luciferase reaction (Beyotime) and an EnSpire multimode plate reader (PerkinElmer) according to the instruction. At least 10 oocytes were added into 30 μL lysis buffer and mixed with 100 μL ATP working solution before detection, and ATP content was calculated using a standard curve.

### 2.8 Single cell RNA sequencing of oocytes

Following the manufacturer's guidelines, the total RNA extracted from MII eggs underwent reverse transcription into cDNA. The resultant cDNA was then fully amplified utilising the Discover‐scTM WTA Kit V2 (N711, Vazyme). The amplified cDNA product was subsequently purified using Aliquot VAHTS DNA Clean Beads, and its quality was assessed using the Agilent 2100 Bioanalyzer. A library was constructed utilising the TruePrep DNA Library Prep Kit V2 for Illumina (TD503, Vazyme), incorporating 1 ng of the cDNA. Subsequently, the library underwent sequencing, which was carried out by Annoroad Gene Technology. DESeq2 was employed for the identification of differentially expressed genes (DEG) using the OmicShare online platform. DEG were defined based on a Foldchange>2 and *p* < 0.05 threshold for the analysis of young blastocysts, while a Foldchange>1.5 and *p* < 0.05 threshold was applied for the analysis of aged oocytes. Additionally, Kyoto Encyclopedia of Genes and Genomes (KEGG) and Gene Ontology (GO) analyses were conducted using the OmicShare online platform. For the hub gene analysis, the DEG were imported to construct protein–protein interaction (PPI) networks through the STRING website, followed by using Cytoscape software to identify hub genes using the degree method.

### 2.9 Molecular docking

The molecular configuration of EGCG (DB12116) was acquired from the Zinc database, while the Arf6 structural model employed for the docking process was sourced from AlphaFold (AF‐P62331‐F1). The docking procedure was meticulously executed using Autodock software, and subsequently, the 3‐dimensional interaction pocket between Arf6 and EGCG was analysed and exhibited utilising Pymol software. The detailed two‐dimensional interactions between EGCG and the active pocket of Arf6 were analysed using LigPlot software.

### 2.10 Statistical Analysis

Statistical analyses were performed using GraphPad software. Experiments were repeated at least three times. Group comparisons employed unpaired *t*‐test or one‐way ANOVA. Significance was denoted as follows: *p < 0.05; **p < 0.01; ***p < 0.001; ****p < 0.0001.

## 3 RESULTS

### 3.1 EGCG supplementation augments the maturation of aged oocytes in vitro

**FIGURE 1:**
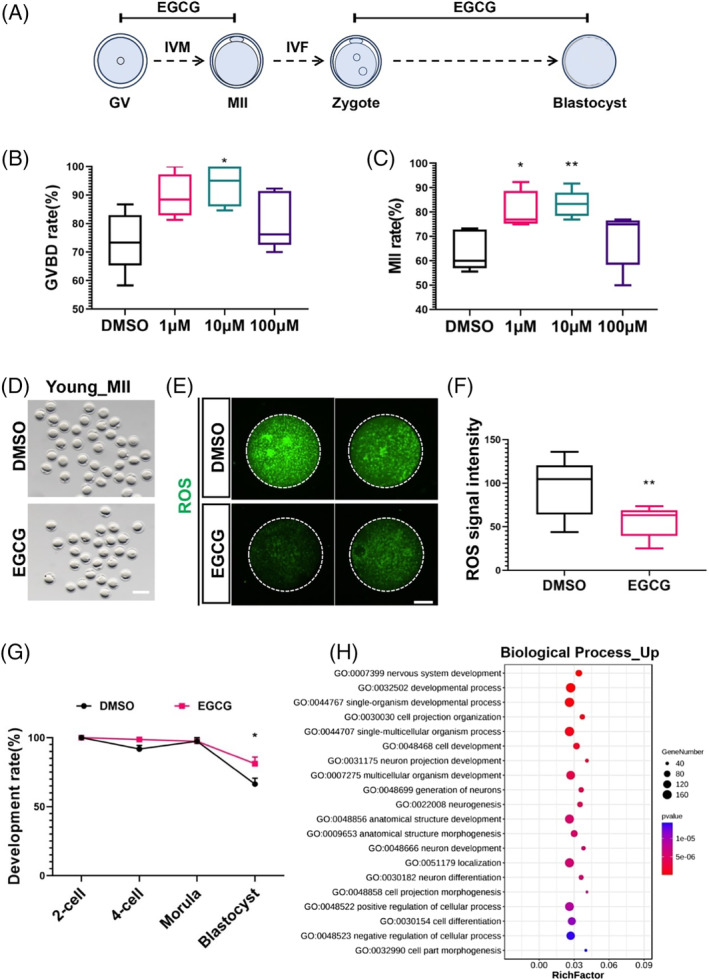
Treating oocytes from young mice with EGCG improves oocyte maturation and early embryonic development. (A) A diagram indicating the EGCG treatment procedure in the process of in vitro maturation (IVM) and in vitro fertilisation (IVF). (B) The rate of geminal vesical breakdown (GVBD) under different concentrations of EGCG. (C) The rate of metaphase II (MII) oocytes under different concentrations of EGCG. The rates of GVBD (B) and MII (C) were observed after cultivation for 16 h with EGCG under different concentrations. About 70 oocytes were used for analyses in each group and the experiments were repeated five times. (D) Representative images of young MII oocytes cultured in vitro with 10 μM EGCG. Scale bar, 100 μm. (E) Representative images of ROS fluorescence (green) in DMSO and 10 μM EGCG‐treated MII oocytes. Scale bar, 20 μm. (F) Quantification of ROS fluorescent intensity in DMSO (*n* = 6) and EGCG‐treated (*n* = 10) oocytes. (G) The development rate of embryos at different development stage with or without 10 μM EGCG treatment. About 80 embryos were used for analyses in each group and the experiments were repeated four times. (H) Gene ontology analysis of the blastocysts between DMSO and EGCG treated group. Four MII oocytes were used separately for the RNA‐seq analysis. One‐way ANOVA analysis was used for (B) and (C). A two‐tailed *t*‐test was used for statistical analysis in (F) and (G). Data are presented as mean ± SEM. *p < 0.05; **p < 0.01.

We initiated an exhaustive investigation that commenced by utilising comparatively youthful oocytes to determine an optimal working concentration, taking into account the relatively restricted accessibility of aged oocytes. The GV oocytes were cultivated in M16 medium, being exposed to varying concentrations of EGCG (0, 1, 10 and 100 μM) (Figure 1A). After a 16‐hour incubation period, a remarkable trend emerged: oocytes treated with 1 μM and notably 10 μM EGCG exhibited considerably enhanced rates of germinal vesicle breakdown (GVBD) and maturation into metaphase II (MII) oocytes (Figure 1B,C). Subsequently, the matured MII oocytes treated with 10 μM EGCG underwent ROS analysis, unveiling a significant reduction in ROS levels post EGCG administration (Figure 1D–F). Encouragingly, fertilised zygotes subjected to EGCG treatment demonstrated marked improvements in blastocyst development (Figure 1A,G and Figure S1A). A deeper dive into these blastocysts via single‐cell RNA sequencing (RNA‐seq) divulged numerous developmental enhancements stemming from EGCG treatment (Figure 1H and Figure S1B,C).

**FIGURE 2:**
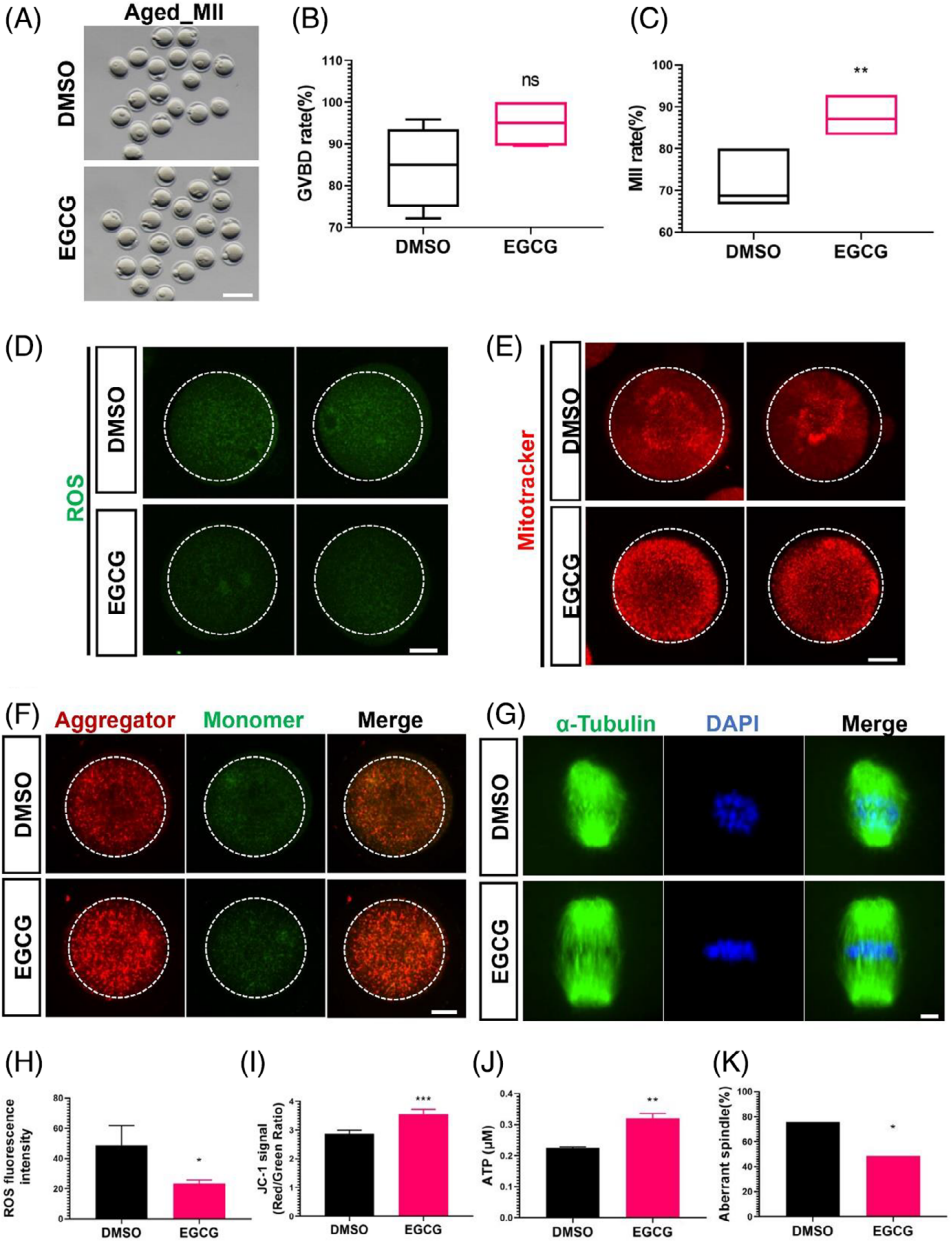
EGCG can significantly improve the in vitro maturation rate of aged oocytes. (A) Representative images of old MII oocytes cultured in vitro with or without 10 μM EGCG. Scale bar, 100 μm. (B) The rate of geminal vesical breakdown (GVBD) with or without 10 μM EGCG in aged oocytes. (C) The rate of metaphase II (MII) oocytes with or without 10 μM EGCG in aged oocytes. The rates of GVBD (B) and MII (C) were observed after cultivation for 16 h with control DMSO or 10 μM EGCG. About 70 embryos were used for analyses in each group and the experiments were repeated four times. (D) Representative images of ROS fluorescence (green) in DMSO and 10 μM EGCG‐treated MII oocytes. Scale bar, 20 μm. (E) Confocal micrographs showing the distribution of mitochondria in oocytes treated with or without EGCG. Mitochondria were stained with Mito‐Tracker. Scale bar, 20 μm. (F) Confocal images showing the mitochondrial membrane potential (MMP), as determined by probe JC‐1, for oocytes treated with or without EGCG. Scale bar, 20 μm.(G) Representative images of spindle morphology in oocytes from aged mice with or without EGCG treatment. Spindles were stained with α‐tubulin antibody (green), and chromosomes were counterstained with DAPI (blue). Scale bar, 10 μm. (H) Quantification of ROS fluorescent intensity in DMSO (*n* = 24) and EGCG‐treated (*n* = 21) oocytes. (I) Quantification of MMP (red/green) as determined by staining the MII oocytes (*n* = 21) with JC‐1. (J) Quantification of the adenosine triphosphate (ATP) content for the oocyte treated with or without EGCG and the experiments were repeated three times. Data are presented as means ± SEM with two‐tailed unpaired Student's *t*‐tests in (B, C) and (H–J). Chi‐Squared test was applied in (K) and about 40 oocytes were used for analyses in each group. *p < 0.05; **p < 0.01; ***p < 0.001, ns: no significance.

Buoyed by these compelling results, we set our sights on the 10 μM EGCG concentration for further investigations, owing to its profound propensity to enhance both oocyte maturation and development potential. Astonishingly, this same concentration demonstrated a notable maturation rate boost in the context of maternally aged mouse oocytes (Figure 2A–C).

### 3.2 EGCG supplementation improves the quality of aged oocytes

As expected, EGCG, when administered at the optimal concentration, exhibits a positive impact on the maturation rate of maternally aged oocytes. To delve deeper into its advantageous effects, we conducted an extensive investigation. The free radical theory of ageing posits that ROS play a central role in inducing oxidative stress and cellular damage, which contributes to the structural and functional alterations characteristic of cellular senescence and ageing.^8^, ^10^, ^12^, ^26^ To analyse the ROS level after EGCG treatment, we employed GV oocytes obtained from aged female mice and subjected them to cultivation in medium, both with and without the supplementation of 10 μM EGCG, for a duration of 16 h, allowing the GV oocytes to mature to the MII phase. Subsequent analysis unveiled a substantial reduction in ROS levels within aged MII oocytes treated with 10 μM EGCG (Figure 2D,H), aligning seamlessly with EGCG's intrinsic antioxidant properties.

The correlation between mitochondria and the deterioration of oocyte quality during ageing is well‐established in both mammalian models and humans.^13^, ^27^ Oocytes with the poor developmental potential display aberrant mitochondrial distribution, reduced mitochondrial membrane potential (MMP) and lower ATP levels.^13^, ^27^, ^28^ We then aimed to assess whether EGCG could ameliorate age‐related mitochondrial dysfunction. To visualise mitochondrial dynamics, the Mito‐Tracker staining was employed to observe the distribution of mitochondrion in aged MII oocytes. Notably, EGCG demonstrated the capacity to rectify asymmetrical mitochondrial distribution pattern (Figure 2E). Mitochondrial membrane potential was then assessed through JC‐1 staining, calculating the red‐to‐green fluorescence intensity ratio. Remarkably, the MMP index of the EGCG treatment group exhibited a significant increase compared to the control DMSO group (Figure 2F,I). Furthermore, quantitative analysis of ATP levels revealed a substantial enhancement following EGCG treatment in aged oocytes (Figure 2J).

Reproductive ageing also affects chromosome structures and meiotic spindle，leading to occurrence of aneuploidy.^29^, ^30^ We also embarked on an in‐depth comparison of spindle morphology and chromosome alignment among MII oocytes. A spectrum of severe abnormalities was observed within aged oocytes, encompassing spindle elongation, lack of discernible poles, and chromosome misalignment,^31^ while EGCG treatment obviously improved the spindle abnormity in aged oocytes (Figure 2G,K). These comprehensive analyses thus underscore the potential of EGCG to elevate both the nuclear and cytoplasmic quality of aged oocytes, consequently leading to the augmentation of maturation rates within this specific population.

### 3.3 EGCG elevates the maturation of aged oocytes in vivo

**FIGURE 3:**
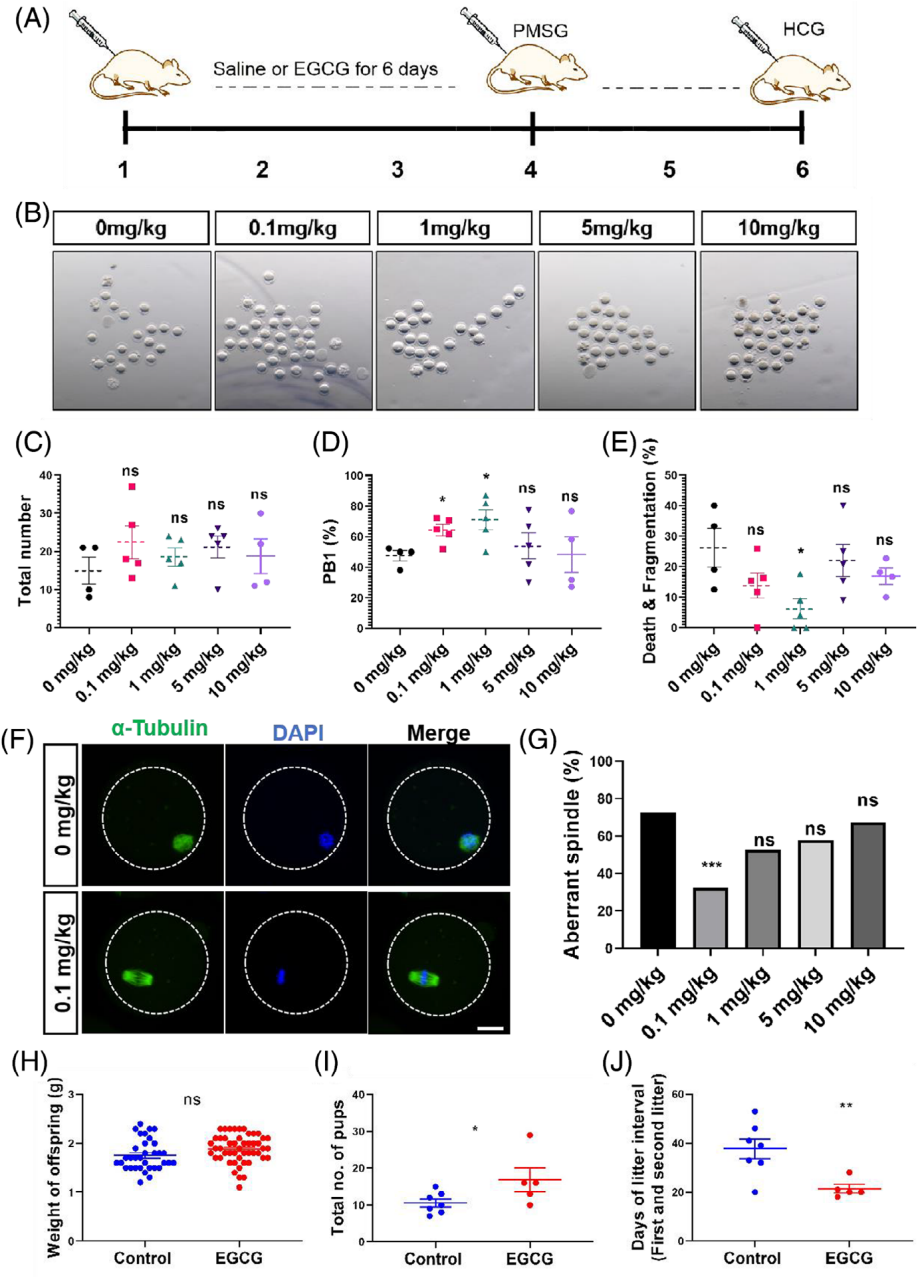
EGCG can significantly improve the in vivo maturation rate of oocytes in aged mice. (A) Schematic of in vivo injection of saline or EGCG to aged mice. Five mice were included in each group through intraperitoneal injection. Five aged females were successfully super‐ovulated in the 0.1, 1 and 5 mg/kg EGCG‐treated groups, whereas only four out of five females were super‐ovulated in the 0 and 10 mg/kg EGCG‐treated groups. (B) Representative images of super‐ovulated MII oocytes from aged mice with 0, 0.1, 1, 5 and 10 mg/kg EGCG treatment. (C) Quantification of the total number of MII oocytes in different EGCG‐treated mice. (D) Quantitative analysis of first polar body (PB1) extrusion rate in the presence of 0, 0.1, 1, 5 and 10 mg/kg EGCG treatment. (E) Quantifications of the death and fragmentation rate of MII oocytes in the presence of 0, 0.1, 1, 5 and 10 mg/kg EGCG treatment. (F) Representative images of spindle morphology in super‐ovulated MII oocytes from aged mice with or without EGCG treatment. Spindles were stained with α‐tubulin antibody (green), and chromosomes were counterstained with DAPI (blue). Scale bar, 20 μm. (G) Quantification of oocytes with abnormal spindle under different EGCG concentrations by Chi‐Squared test. At least 20 oocytes were used in each group for the analysis. (H) Quantification of the weight of offspring in control (*n* = 34) and 0.1 mg/kg EGCG‐treated (*n* = 49) mice. (I) Quantification of the total number of pups per female in control (*n* = 7) and 0.1 mg/kg EGCG‐treated (*n* = 5) mice. (J) Quantification of the days of litter interval (first and second litter) in control (*n* = 7) and 0.1 mg/kg EGCG‐treated (*n* = 5) mice. Data are presented as means ± SEM. *p < 0.05; **p < 0.01, ns: no significant difference. Two‐tailed unpaired Student's *t*‐tests.

To investigate the impact of EGCG on the maturation and quality of aged oocytes in vivo, female mice aged 9–10 months, respectively received intraperitoneal injections of EGCG at doses of 0, 0.1, 1, 5 and 10 mg/kg/d for 6 days (Figure 3A). Superovulation was induced from the fourth day to assess the influence of various EGCG concentrations on the quantity and quality of MII oocytes (Figure 3A). No significant differences were observed in body weight, ovary/body weight and hormone levels among the groups following superovulation (Figure S2A–D). While the total number of oocytes obtained through hormone superovulation showed no substantial disparity across the groups (Figure 3B,C), injection of 0.1 and 1 mg/kg EGCG resulted in a noteworthy increase in first polar body (PB1) extrusion rate (Figure 3B,D), and the 1 mg/kg EGCG injection exhibited a significant reduction in oocyte fragmentation rate (Figure 3B,E). Furthermore, fluorescence staining of collected MII oocytes from each group revealed a considerable decrease in the proportion of abnormal spindles in the 0.1 mg/kg EGCG injection group (Figure 3F,G). These findings collectively indicate EGCG's capacity to enhance the quality of aged oocytes maturation in vivo.

Considering that the decline in oocyte quality stands as a key contributor to age‐related infertility, we formulated the hypothesis that EGCG might improve fertility in aged mice by enhancing oocyte quality. Following a six‐day regimen of intraperitoneal 0.1 mg/kg EGCG administration, aged female mice were paired with male mice of reproductive age for a span of 3 months. To assess the efficacy of EGCG in enhancing fertility among aged mice, several fertility indices were meticulously evaluated. The outcomes revealed no significant disparities in total litters per female and the number of pups per litter subsequent to EGCG treatment (Figure S2E,F). However, a remarkable increase in the overall number of offspring and a reduction in the days of litter interval (pertaining to the first and second litters) were conspicuously observed in the EGCG‐administered group, while the offspring's body weight displayed no significant alterations (Figure 3H–J). These findings further underscore the promising potential of EGCG to enhance fertility in ageing mice.

### 3.4 Effect of EGCG supplementation on transcriptome profiling of aged oocytes

**FIGURE 4:**
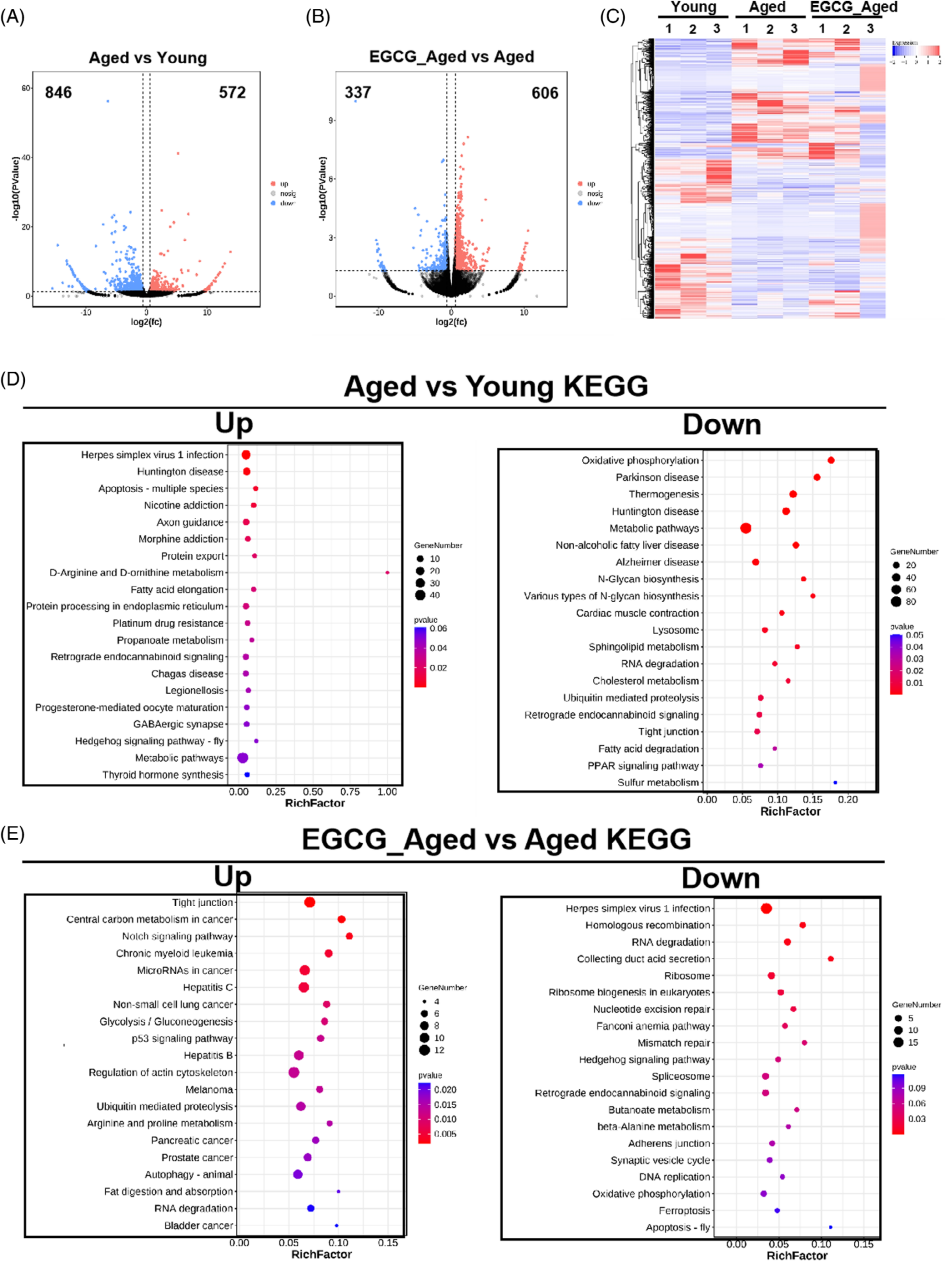
Transcriptome analysis of EGCG‐treated oocytes from aged mice. (A) Volcanic plot documenting differentially expressed genes (DEGs) between young and aged MII oocytes. Red colour: Up‐regulated; Blue colour: Down‐regulated. (B) Volcanic plot documenting differentially expressed genes (DEGs) between aged and EGCG‐treated aged MII oocytes. Red colour: Up‐regulated; Blue colour: Down‐regulated. (C) Heatmap documenting differentially expressed genes (DEGs) between young, aged and EGCG‐treated aged oocytes. (D) Kyoto Encyclopedia of Genes and Genomes (KEGG) enrichment analysis between young and aged MII oocytes. (E) Kyoto Encyclopedia of Genes and Genomes (KEGG) enrichment analysis between aged and EGCG‐treated aged MII oocytes.

To gain deeper insights into the potential mechanisms underlying the impact of EGCG on the quality of aged oocytes, we conducted single cell RNA‐seq analysis on MII oocytes obtained from young mice, aged mice, and EGCG‐treated aged mice. The resulting heatmap and volcano plot data revealed substantial differences (*p* < 0.05; Fold change>1.5) in the transcriptome profiles of aged oocytes compared to young oocytes (Figure 4A,C). Specifically, 572 differentially expressed genes (DEGs) were up‐regulated, and 846 DEGs were down‐regulated in aged oocytes (Figure 4A,C and Figure S3A,C). Furthermore, EGCG treatment displayed 606 up‐regulated genes and 337 down‐regulated genes compared with aged oocytes and 868 up‐regulated genes and 902 down‐regulated genes compared with young oocytes (Figure 4B,C and Figure S3A–D).

KEGG analysis of the DEGs unveiled abnormal expression pathways in aged oocytes related to apoptosis, the ubiquitin‐mediated proteolysis and viral infection in comparison to young oocytes (Figure 4D). However, these pathways were restored in EGCG‐treated aged oocytes (Figure 4D,E). Notably, a significant improvement was observed in the actin cytoskeleton regulation pathway and oocyte meiosis pathway of EGCG‐treated aged oocytes, when compared to aged and young oocytes (Figure 4E and Figure S3E), suggesting that EGCG might be involved in the regulation of oocyte cytoskeleton structure and further analysis need to be conducted.

### 3.5 EGCG may regulate the actin cytoskeleton of aged oocytes through Arf6

**FIGURE 5:**
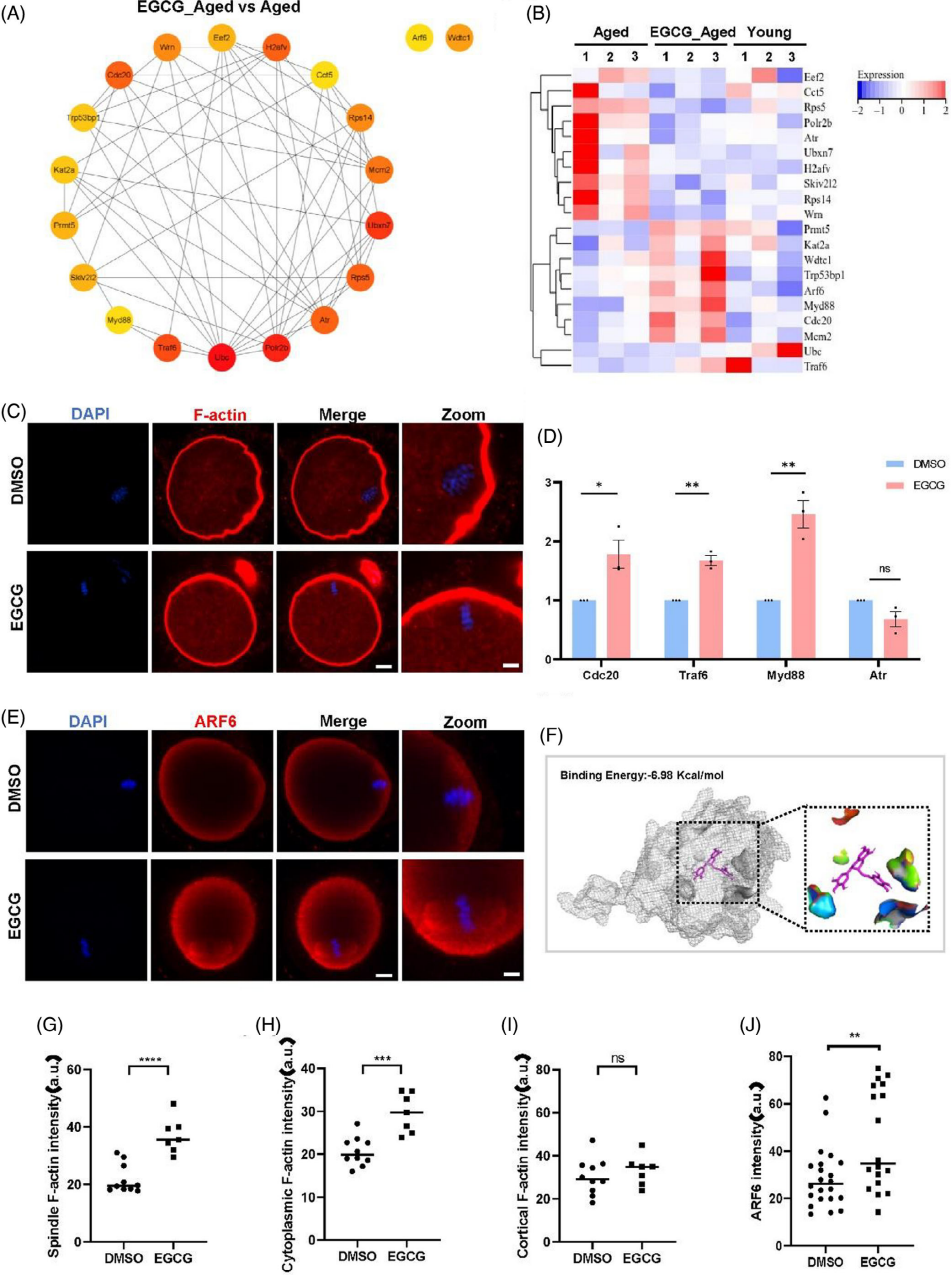
EGCG may regulate the actin cytoskeleton of aged oocytes through Arf6. (A) Protein–protein interaction network (PPI) of top 20 hub genes between aged and EGCG‐treated aged oocytes. (B) Heatmap documenting the top 20 hub genes between aged and EGCG‐treated aged oocytes. (C) Representative images of F‐actin in oocytes from aged mice with (*n* = 7) or without (*n* = 10) EGCG treatment. F‐actin were stained with Rhodamine (red), and chromosomes were counterstained with DAPI (blue). Scale bar, 10 μm. (D) Validation of RNA‐seq data by qPCR for at least three independent replicates. (E) Representative images of the distribution of Arf6 in oocytes from aged mice with or without EGCG treatment. Arf6 were stained with anti‐Arf6 antibody (red), and chromosomes were counterstained with DAPI (blue). Scale bar, 10 μm. (F) Molecular docking pattern of EGCG and Arf6. (G) Quantification of spindle F‐Actin fluorescent intensity in aged (*n* = 10) and EGCG‐treated (*n* = 7) aged oocytes. (H) Quantification of cytoplasmic F‐actin fluorescent intensity in aged (*n* = 10) and EGCG‐treated (*n* = 7) aged oocytes. (I) Quantification of cortical F‐actin fluorescent intensity in aged (*n* = 10) and EGCG‐treated (*n* = 7) aged oocytes. (J) Quantification of Arf6 fluorescent intensity around spindle in aged (*n* = 22) and EGCG‐treated (*n* = 18) aged oocytes. Data are displayed as means ± SEM. *p < 0.05; **p < 0.01; ****p < 0.0001, ns: no significant difference. Two‐tailed unpaired Student's *t*‐tests.

By effectively leveraging the STRING database and employing Cytoscape software, we intricately pieced together PPI networks that encompassed the differentially expressed genes within aged oocytes and EGCG‐treated aged oocytes. Subsequently, a meticulous analysis led us to pinpoint 20 hub genes of particular significance (Figure 5A and Table S1). Among this set, 10 DEGs experienced up‐regulation, while the remaining 10 DEGs displayed down‐regulation (Figure 5B). Furthermore, the validation of transcriptome profiling was carried out via quantitative real‐time PCR for a selection of randomly chosen genes compared to GAPDH (Figure 5D). These DEGs are implicated in various critical processes, encompassing oocyte cytoskeleton dynamics (Arf6),^32^ ovulation (Myd88),^33^ cell cycle regulation (Cdc20),^34^, ^35^ oocyte and embryo development (Atr, Rps14, Eef2),^36^, ^37^, ^38^, ^39^ oxidative stress (Wrn),^40^ DNA damage response (Trp53bp1),^41^ among others.

Aged oocytes exhibit a disturbance in spindle F‐actin, which in turn exacerbates the premature separation of sister chromatids and leads to egg aneuploidy.^42^ Notably, as illustrated by the upregulation of the actin cytoskeleton regulatory gene in aged oocytes following EGCG treatment (Figure 4E), we embarked on investigating its potential role in regulating F‐actin. Intriguingly, the application of EGCG indeed led to a substantial enhancement in the expression of F‐actin, especially around the spindle region (Figure 5C,G–I), underscoring the potential of EGCG to mitigate oocyte aneuploidy, at least in part, through the regulation of F‐actin.

In order to unravel the mechanism underlying the regulation of F‐actin by EGCG, our focus turned towards an upregulated hub gene, Arf6 (ADP‐ribosylation factor 6). Arf6, a small GTPase within the Ras‐related G protein family, holds a pivotal role in orchestrating vesicular traffic and actin remodelling.^43^ Existing research has highlighted Arf6's importance on actin nucleation factors in mouse oocytes, affecting actin‐mediated spindle movement and assembly, and polar body extrusion.^32^ And a prior study has demonstrated that EGCG could potentially interact with the cytohesin‐1–Arf6 complex, as revealed by molecular docking analyses.^44^ Guided by these insights, we proposed the hypothesis that EGCG could modulate actin nucleation factors and promote spindle F‐actin assembly through regulating Arf6. Subsequently, we examined the expression of Arf6 in MII oocytes treated with EGCG from aged mice. Impressively, a comparable localisation pattern between Arf6 and F‐actin was observed, especially encompassing the spindle region (Figure 5C,E). Remarkably, fluorescence quantitative analysis unveiled a significant elevation in the level of Arf6 within EGCG‐treated aged oocytes, especially around the spindle (Figure 5E,J), further indicating the importance of Arf6 to enhance spindle associated F‐actin. In addition, we obtained the structural information of Arf6 from the AlphaFold database and EGCG from the Zinc database to execute molecular docking, exploring the potential interaction between the ligand (EGCG) and the receptor (Arf6). The docking analysis underscored a conceivable interaction between Arf6 and EGCG, displaying a binding energy of −6.98 Kcal/mol (Figure 5F and Figure S4).

In summary, our findings collectively suggest an additional mechanism through which EGCG can enhance the expression of Arf6, thus potentially participating in the assembly process of spindle F‐actin within aged mouse oocytes. This plausible mechanism holds the potential to mitigate the increased aneuploidy rate triggered by the disruption of spindle F‐actin in aged oocytes, a significant characteristic of reproductive ageing.

## 4 DISCUSSION

**FIGURE 6:**
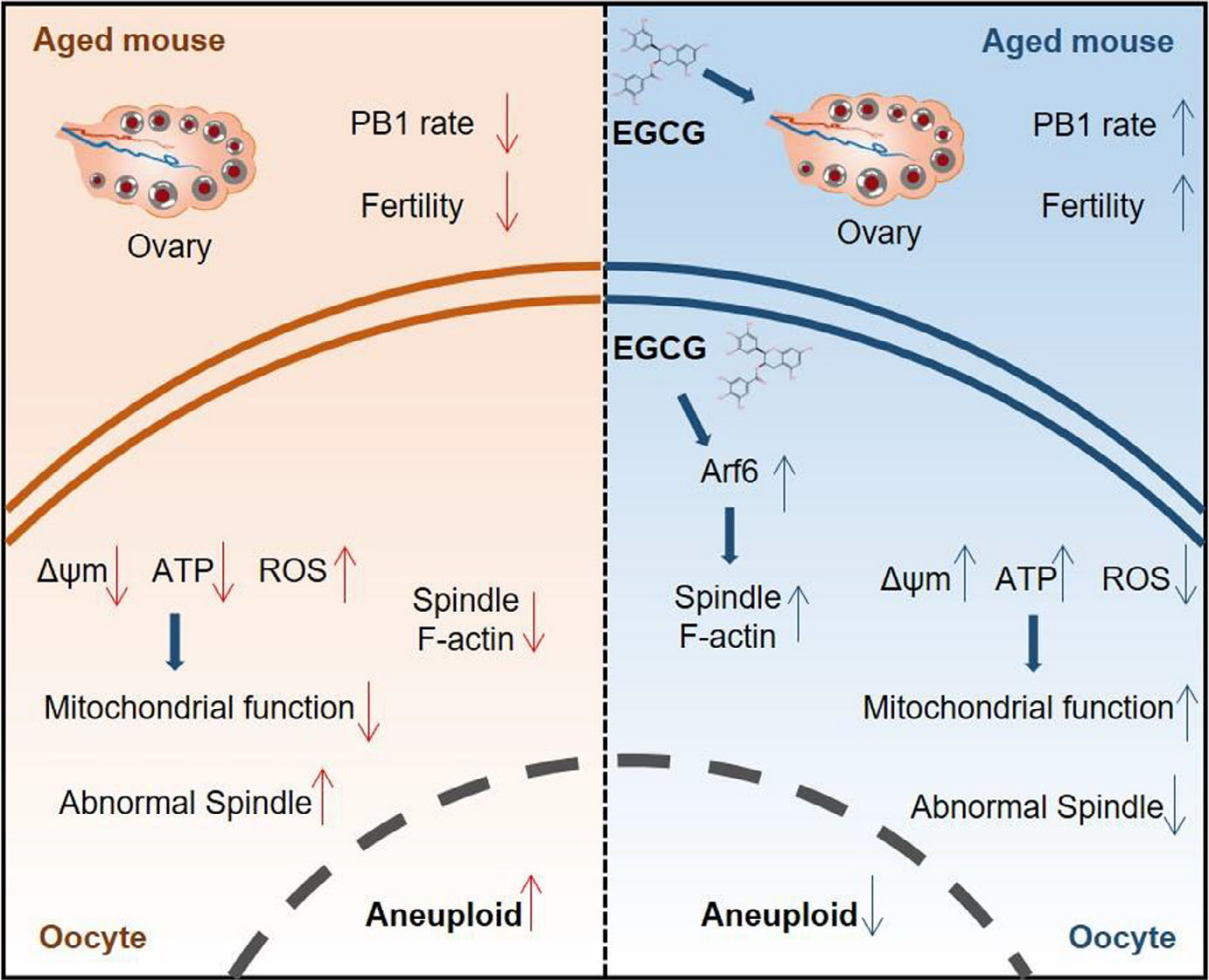
A diagram indicates the effect of EGCG administration on improved quality of aged oocytes.

Previous experiments on animal models such as pigs, cattle and mouse have confirmed that the antioxidant activity of EGCG has the potential to improve oxidative stress‐related infertility.^20^, ^21^, ^22^, ^23^ However, there is no direct evidence to date that the antioxidant activity of EGCG has any effect on aged oocytes. Here, we administrated EGCG to aged oocytes both in vitro and in vivo and found remarkable improvements in oocyte quality and female fertility. In particular, EGCG, when administered at a moderate concentration, exhibited the ability to effectively curtail the production of ROS, thereby mitigating oxidative stress. Moreover, EGCG exhibited regulatory effects on mitochondrial activity and distribution, while simultaneously refining spindle morphology. What's more, our study unveiled a potential impact of EGCG on Arf6, which facilitated the assembly of F‐actin especially around the spindle in aged oocytes. This intricate process holds the potential to counteract the age‐related increase in aneuploidy (Figure 6).

Given the rising trend of delayed pregnancy, the need to identify interventions that can preserve or extend human fertility has become exceedingly crucial. Presently, there are no clinically viable approaches to effectively counteract or reverse the ovarian and uterine dysfunction linked with advanced age.^45^ Nevertheless, significant progress has been achieved in the realm of senotherapy in recent years. Notable examples include quercetin,^31^ IGF2,^46^, ^47^ NAD and its precursors,^45^, ^48^ resveratrol,^49^ melatonin,^50^, ^51^ coenzyme Q10,^52^ and more. These interventions have demonstrated the potential to enhance oocyte quality and alleviate infertility, particularly in aged rodent models. As an antioxidant, EGCG at relatively low concentrations has shown a protective effect on oocyte quality across various species.^20^, ^23^, ^25^ However, higher concentrations of EGCG can lead to pro‐oxidative effects. For instance, microinjection of 1 mM EGCG inhibited progesterone‐induced oocyte maturation in Xenopus laevis,^53^ and treatment with 25–50 mM EGCG significantly increased apoptosis in mouse blastocyst cells.^54^ These findings underscore the importance of using an appropriate concentration to achieve the desired pharmacological effect of the drug.

Aneuploidy within eggs stands as a prominent factor contributing to infertility, miscarriages and congenital disorders. Notably, the incidence of aneuploidy in human eggs rises with maternal age, surpassing 50% in eggs from women aged 35 years.^55^, ^56^ A recent investigation has implicated the disruption of spindle‐associated F‐actin as a pivotal factor in female reproductive ageing, exacerbating the occurrence of aneuploidy.^42^ Indeed, actin plays multifaceted roles during meiosis.^57^ For instance, it orchestrates accurate alignment and segregation of chromosomes,^58^ regulates microtubule‐coordinated spindle localisation^59^ and facilitates the long‐range transportation of vesicles in mouse oocytes.^60^ With advancing reproductive age, the depletion of cohesive proteins and the disruption of spindle‐associated F‐actin collectively diminish spindle actin‐mediated resistance to microtubule‐induced tension, culminating in premature separation of sister chromatids and subsequent aneuploidy.^42^

The transcriptomic evidence pointing to the upregulation of the cytoskeleton regulation pathway following EGCG treatment prompted us to focus on the expression of F‐actin around the spindle, wherein we indeed observed an upregulation. It's worth noting that a small molecular weight GTPase, Arf6, exhibited upregulation in EGCG‐treated oocytes. Arf6 plays a pivotal role in diverse cellular processes, including cytoplasmic division, phagocytosis, and cell migration by orchestrating the recombination of actin filaments.^43^, ^61^ In mouse oocyte, Arf6 knockout can lead to abnormal assembly of F‐actin, failure movement of spindle and destruction of spindle formation.^32^ Interestingly, our findings showcased that Arf6 exhibited co‐localisation with spindle F‐actin, while the expression of Arf6 notably increased in aged mouse oocytes treated with EGCG. Additionally, molecular docking outcomes indicated the potential binding of EGCG to Arf6, although further exploration is required to confirm this interaction and to fully understand how EGCG enhances Arf6 activity. In any case, our data hints at the possibility that EGCG might enhance the expression of Arf6 through direct interaction, consequently fostering the assembly of spindle F‐actin and thereby safeguarding mouse oocytes from age‐related aneuploidy.

In conclusion, our study lends support to the proposition that EGCG holds the capability to enhance oocyte quality in aged mice. This investigation not only validated the protective role of EGCG against oxidative stress in ageing oocytes but also unveiled a potential avenue for mitigating the aneuploidy rate in these oocytes by improving the spindle morphology and modulating spindle F‐actin via Arf6. By shedding light on the multifaceted mechanisms through which EGCG impacts oocyte quality, our work not only contributes to the comprehension of EGCG's role in bolstering oocyte health but also underscores its potential as a therapeutic intervention for addressing fertility challenges associated with advanced age.

## AUTHOR CONTRIBUTIONS

Honghui Zhang performed the majority of experiments; Wei Su conducted the transcriptome verification work; RuSong Zhao helped with the transcriptome analysis; Mei Li and ShiGang Zhao provided technical and theoretical help; HongHui Zhang and Wei Su wrote the manuscript; Zi‐Jiang Chen and Han Zhao revised the manuscript. All authors discussed the results and commented on the manuscript.

## FUNDING INFORMATION

The work was supported by the National Key Research and Development Program of China (2021YFC2700400), the Basic Science Center Program of NSFC (31988101), CAMS Innovation Fund for Medical Sciences (2021‐I2M‐5‐001), the National Natural Science Foundation of China (82192874, 82071606, 82171842), Shandong Provincial Key Research and Development Program (2020ZLYS02), the Taishan Scholars Program of Shandong Province (ts20190988), the Innovative research team of high‐level local universities in Shanghai (SHSMU‐ZLCX20210200) and the Fundamental Research Funds of Shandong University.

## CONFLICT OF INTEREST STATEMENT

The authors declare that they have no conflict of interest.

## Supporting information


**DATA S1:** Supporting Information.

## Data Availability

Data can be obtained from the corresponding author under reasonable request.
