# Supplementary material for: Epigallocatechin‐3‐gallate improves the quality of maternally aged oocytes
Source: Cell Prolif. 2023 Nov 27;57(4):e13575. doi: 10.1111/cpr.13575 (PMC10984106; doi:10.1111/cpr.13575)
Supplement: Supplementary file 1 — DATA S1: Supporting Information. [file CPR-57-e13575-s001.docx]

**Supplementary Material**

**Epigallocatechin-3-gallate improves the quality of maternally aged oocytes**

HongHui Zhang^1,2,3,4^, Wei Su^1,3,4^, RuSong Zhao^1,2,3,4^, Mei Li^1,3,4^, ShiGang Zhao^1,3,4^, Zi-Jiang Chen^1,3,4,5,6^, Han Zhao^1,3,4^*


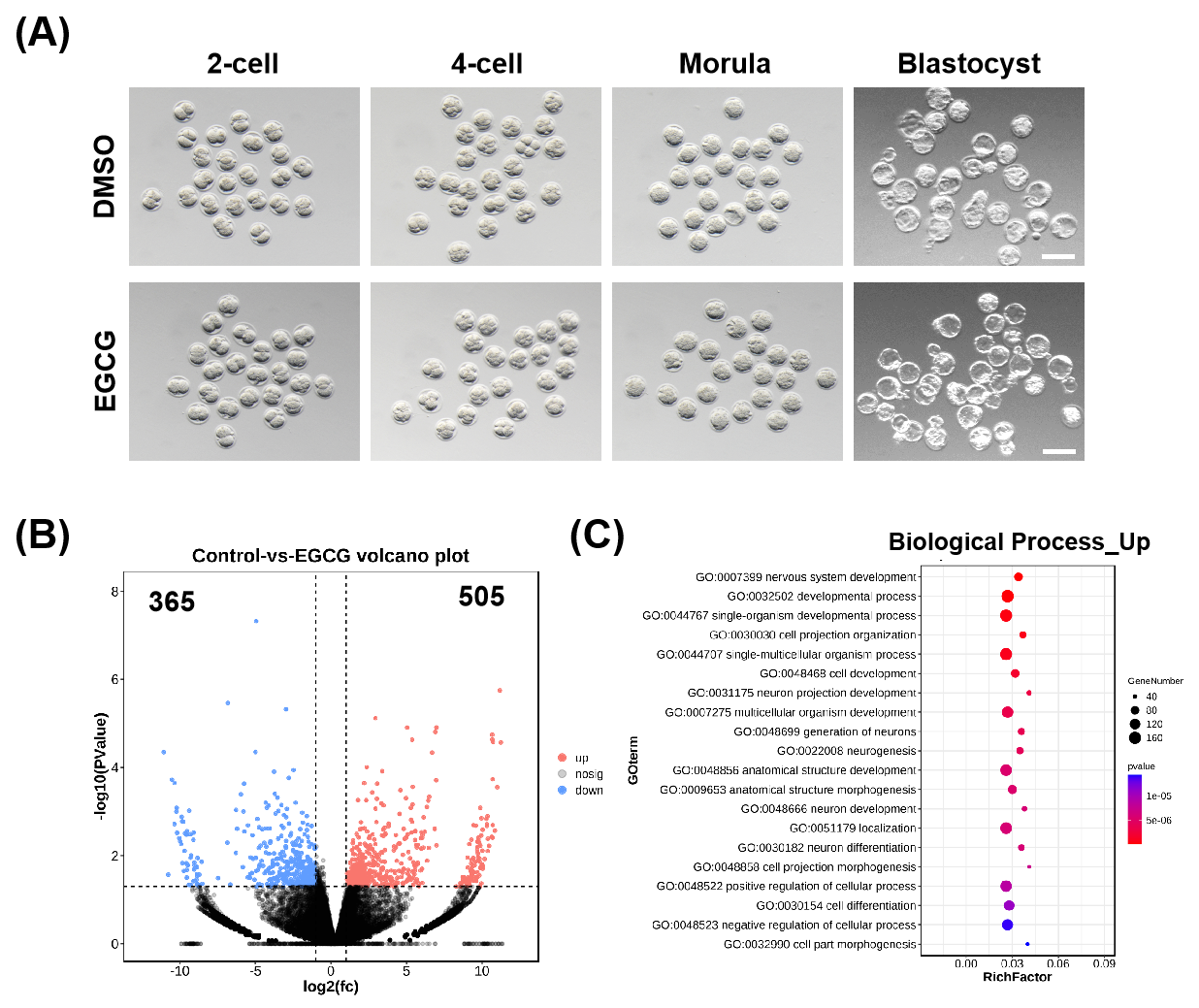


**Supplementary Figure 1. 10μM EGCG can significantly up-regulate development-related pathways in blastocysts.** (A) Representative images of embryos cultured in vitro at the 2-cell, 4-cell, morula and blastocyst stages with or without 10μM EGCG. Scale bar, 100μm. (B) Volcanic plot documenting DEGs from control and 10μM EGCG-treated embryos cultured in vitro at the blastocyst stage. (C) Gene Ontology (Biological Process) enrichment analysis of up-regulated DEGs from control and 10μM EGCG-treated blastocysts.

**
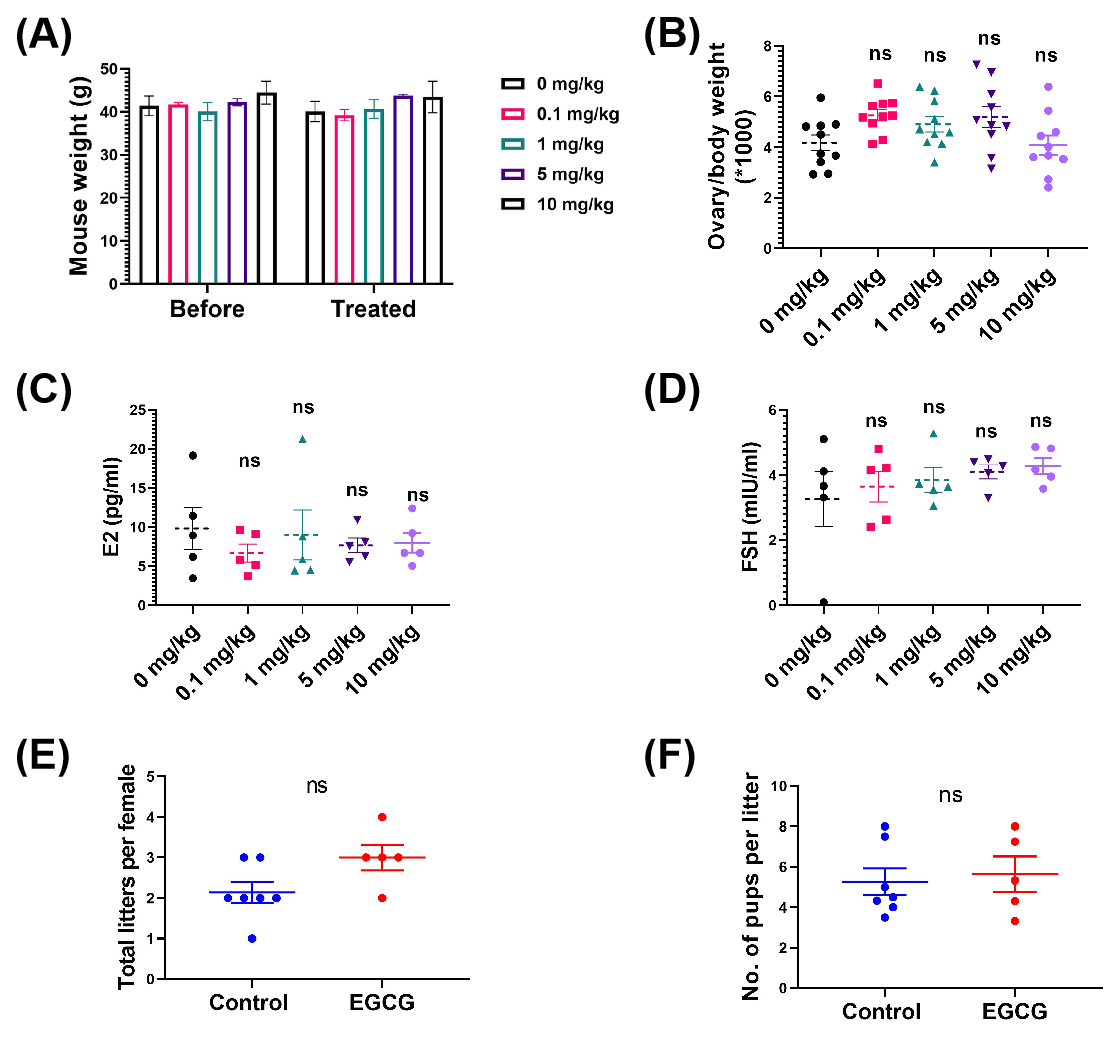
**

**Supplementary Figure 2. EGCG injection in vivo can improve the fertility of aged mice.** (A) Quantification of mouse weight changes in aged mice after 6 days EGCG injection. Two-way ANOVA analysis was used with no significant difference. (B) Quantification of the ovary/body weight (*1000) in aged mice after the presence of 0,0.1,1,5 and 10 mg/ml EGCG treatment. (C) Quantification of the estradiol levels (E2, ng/ml) in aged mice after the presence of 0,0.1,1,5 and 10 mg/ml EGCG treatment. (D) Quantification of the follicle stimulating hormone levels (FSH, ng/ml) in aged mice after the presence of 0,0.1,1,5 and 10 mg/ml EGCG treatment. One-way ANOVA analysis was used for (B-D). (E) Quantification of total litters per female in control (n=7) and 0.1mg/kg EGCG-treated (n=5) mice. (F) Quantification of the number of pups per litter in control (n=7) and 0.1mg/kg EGCG-treated (n=5) mice. Two-tailed unpaired Student’s t-tests in (E-F). Data are displayed as means ± SEM. ns: no significant difference.


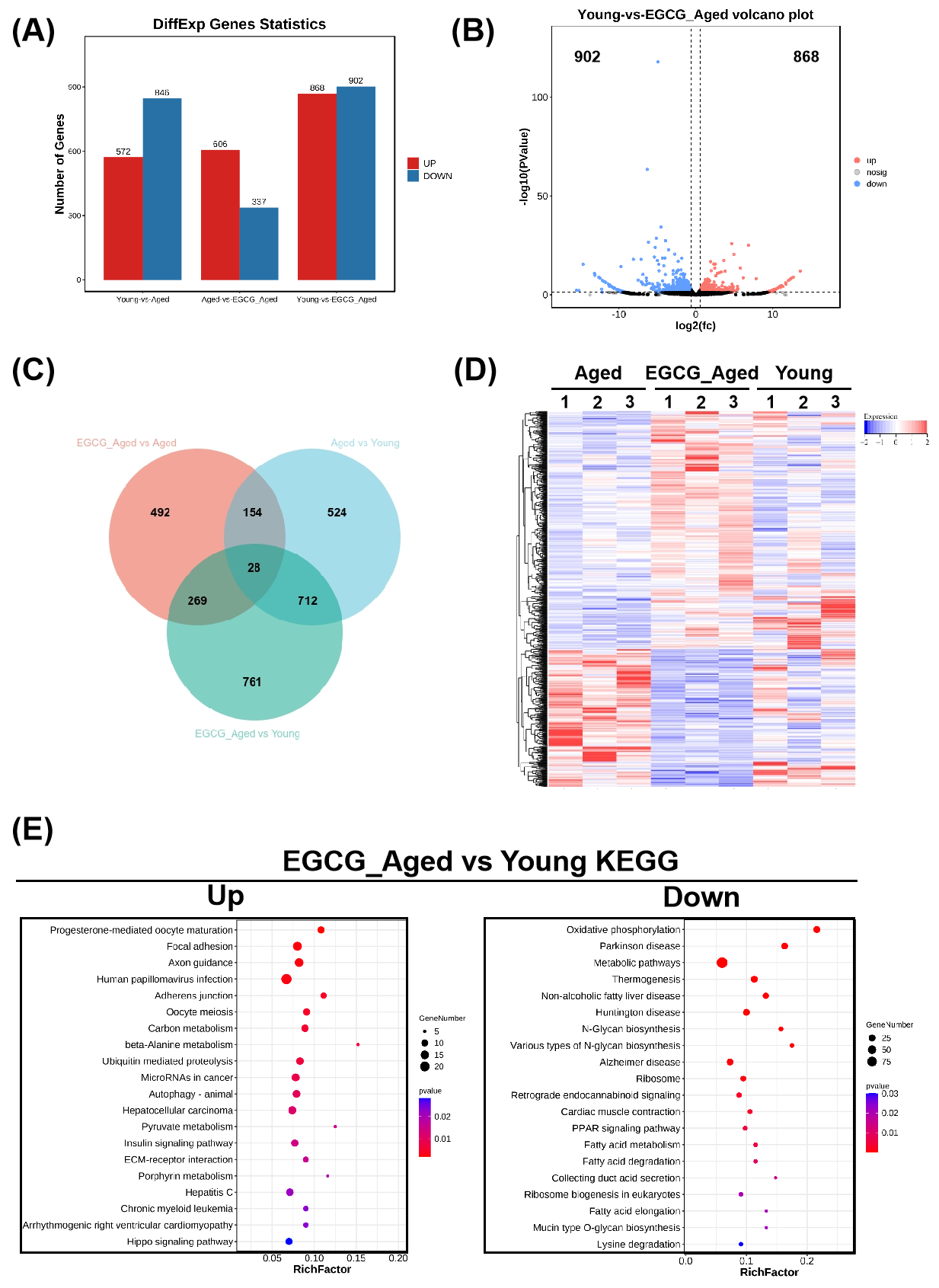


**Supplementary Figure 3. Transcriptome analysis of EGCG-treated oocytes from aged mice.** (A) The histogram showed the number of the up-regulated and down-regulated DEGs between young, aged, and EGCG-treated aged oocytes. (B) Volcanic plot documenting DEGs between young and EGCG-treated aged oocytes. (C) The pie chart shows the number of DEGs between young, aged and EGCG-treated aged oocytes. (D) Heatmap documenting DEGs between young, aged and EGCG-treated aged oocytes. (E) KEGG analysis between young and EGCG-treated aged MII oocytes.


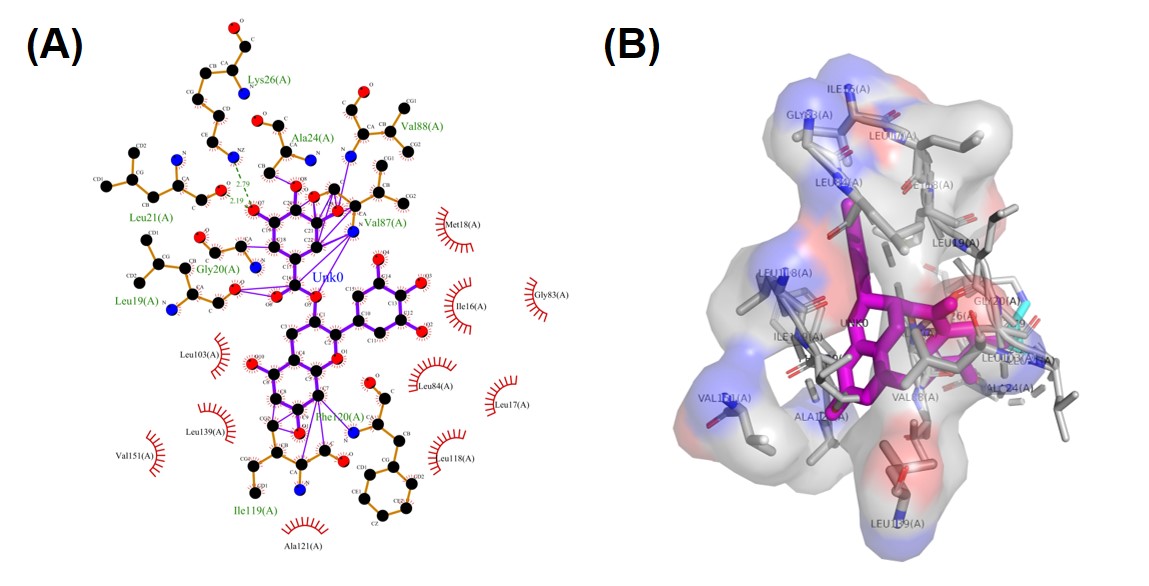


**Supplementary Figure 4. Two-dimensional interactions between EGCG and Arf6.** (A) The interactions between EGCG and Arf6 were analyzed using LigPlot. 'Unk0' represents EGCG. The green lines and labels indicate hydrogen bonds and the corresponding amino acids. The red eyelash figures represent hydrophobic amino acids. (B) The interactions were visualized in three dimensions using PyMol software.

**Table S1.**

**According to the degree method, the top 20 hub genes in the control DMSO and EGCG-treated aged mouse oocytes were ranked by Cytoscape software.**

| **Rank** | **Name** | **Score** |
| --- | --- | --- |
| 1 | Ubc | 62 |
| 2 | Polr2b | 42 |
| 3 | Ubxn7 | 39 |
| 4 | Traf6 | 33 |
| 5 | Rps5 | 31 |
| 5 | H2afv | 31 |
| 5 | Cdc20 | 31 |
| 5 | Atr | 31 |
| 9 | Mcm2 | 30 |
| 10 | Rps14 | 29 |
| 10 | Wrn | 29 |
| 12 | Wdtc1 | 28 |
| 13 | Prmt5 | 27 |
| 13 | Eef2 | 27 |
| 13 | Skiv2l2 | 27 |
| 16 | Kat2a | 26 |
| 16 | Trp53bp1 | 26 |
| 18 | Myd88 | 25 |
| 18 | Arf6 | 25 |
| 18 | Cct5 | 25 |

**Table S2. Primer information of qPCR**

| **Primer name** | **Sequence** |
| --- | --- |
| Cdc20-F | TTCGTGTTCGAGAGCGATTTG |
| Cdc20-R | ACCTTGGAACTAGATTTGCCAG |
| Traf6-F | AAAGCGAGAGATTCTTTCCCTG |
| Traf6-R | ACTGGGGACAATTCACTAGAGC |
| Myd88-F | AGGACAAACGCCGGAACTTTT |
| Myd88-R | GCCGATAGTCTGTCTGTTCTAGT |
| Atr-F | GAATGGGTGAACAATACTGCTGG |
| Atr-R | TTTGGTAGCATACACTGGCGA |
| GAPDH-F | CTGCACCACCAACTGCTTAG |
| GAPDH-R | GGATGCAGGGATGATGTTCT |

**Table S3. Antibodies used for immunofluorescence staining of oocytes**

| **Antibody** | **Source** | **Identifier** |
| --- | --- | --- |
| Anti-α-Tubulin-FITC antibody | Sigma | F2168 |
| ARF6 Polyclonal Antibody | Invitrogen | PA1-093X |
| Rhodamine Phalloidin | Invitrogen | R415 |
